# Revision of encapsulated blebs after trabeculectomy: Long-term comparison of standard bleb needling and modified needling procedure combined with transconjunctival scleral flap sutures

**DOI:** 10.1371/journal.pone.0178099

**Published:** 2017-05-18

**Authors:** P. Laspas, P. D. Culmann, F. H. Grus, V. Prokosch-Willing, A. Poplawksi, N. Pfeiffer, E. M. Hoffmann

**Affiliations:** Department of Ophthalmology, University Medical Center, Johannes Gutenberg- University Mainz, Mainz, Germany; Universita degli Studi di Firenze, ITALY

## Abstract

**Purpose:**

To compare two surgical approaches for treating encapsulated blebs after trabeculectomy with mitomycin C, in terms of the development of intraocular pressure and progression of glaucoma in a long-term follow up: 1. bleb needling alone vs. 2. a combined approach of needling with additional transconjunctival scleral flap sutures, to prevent early ocular hypotony.

**Methods:**

Forty-six patients with failing blebs after trabeculectomy with mitomycin C were enrolled in this study. Patients received either needling revision alone (group 1; n = 23) or a combined needling with additional transconjuctival flap sutures, if intraoperatively the intraocular pressure was estimated to be low (group 2; n = 23). Intraocular pressure (IOP), visual acuity, visual fields, and optic nerve head configuration by means of Heidelberg Retina Tomograph (HRT^®^) were analysed over time. Results from both groups were compared using Mann-Whitney U-test for single timepoints.

**Results:**

IOP did not differ significantly between the two groups during follow-up at three months (P = 0.13), six months (P = 0.12), one year (P = 0.92) and two years (P = 0.57) after surgery. Furthermore, there was no significant difference in the course of glaucoma concerning the optic nerve anatomy between the two groups (Rim Area Change in the Moorfields Regression Analysis of HRT^®^) till two years after surgery (P = 0.289). No functional impairment in visual acuity and visual fields was found in the groups of the study.

**Conclusions:**

Single needling procedure is a standard successful method for restoring the function of encapsulated blebs. Postoperative hypotony represents a possible hazard, which can be minimized by additional transconjunctival flap sutures. Long-term results suggest that this modification is equally effective in lowering the IOP and preventing the progression of glaucoma as the standard needling procedure. To our knowledge this is the first study to investigate the long-term effect of tranconjunctival sutures for the prevention of hypotony.

## Introduction

Lowering of intraocular pressure (IOP) remains the only available therapeutic strategy in the treatment of glaucoma. Trabeculectomy is the gold standard surgical procedure for obtaining an adequate IOP reduction [1]. Modifications in the procedure, such as the introduction of antimetabolites, further improve its long–term outcome [2–4]. However, bleb encapsulation with impairment of the aqueous outflow and increase of IOP remains a frequent postoperative challenge to manage in about 13% of the cases [5, 6].

In most cases, a transconjunctival needling of the bleb is performed, in which the encapsulated bleb is opened and the aquous outflow is restored [7–9]. A dangerous complication of needling is the hypotonic shift [10, 11]. Typical signs of ocular hypotony on the first postoperative days are flattening of the anterior chamber, hyperfiltration, choroidal effusion or choroidal haemorrhage. In the long-term, maculopathy, chorioretinal folds on the posterior pole, tortuosity of the retinal vessels and visual loss may occur [12].

A modification in the standard needling procedure, which has been shown to be successful in preventing early ocular hypotony [13], is the additional placement of transconjunctival scleral flaps during the needling procedure. If the filtration through the bleb seems intraoperatively too much, one or more sutures can easily be placed onto the scleral flap through the intact conjunctiva, until the IOP is regulated. This allows to prevent profound ocular hypotony after the surgery and also to finely adjust the IOP later through their elective removal if needed [14, 15].

In this retrospective study, we present the long-term results of this surgical approach on our initial therapeutic target: IOP regulation and the stabilisation of glaucoma disease. Two-year results of patients who underwent this modified procedure are compared with patients who received a standard bleb needling. The measures of outcome were IOP, the need for additional therapy or revision, any anatomical changes in the optic nerve head, visual acuity and the visual field course. To our knowledge this is the first study to investigate the long-term effect of tranconjunctival sutures for the prevention of hypotony.

## Methods

The study was conducted according to the tenets of the Declaration of Helsinki, and was approved through the local ethics committee of Rhineland-Palatinate. An interinstitutional, retrospective, interventional case series with 46 patients treated at the Department of Ophthalmology, Mainz University Medical Center between 2009 and 2014 was evaluated. All patients had a non-functional encapsulated bleb with high IOP after trabeculectomy with MMC. The attending ophthalmologist indicated a bleb revision in an individualized manner for each patient, taking into consideration the stage and the course of glaucoma and the targeted IOP. The surgical procedure (indication, perspective, dangers) was fully explained to all patients, who gave signed consent.

The study included two groups of 23 patients: “Group 1” underwent revision of the filtering bleb through a single needling procedure, while “Group 2” received a combined procedure of needling with additional transconjunctival scleral flap sutures.

Surgery was performed in the OR under local or general anaesthesia by one surgeon (NP). After disinfection with 1% povidone-iodine solution (Braun, Melsungen, Germany) and intended depression of gaze, the conjunctiva was penetrated with a 14-gauge needle at a considerable distance from the scleral flap, in order to avoid a postoperative leakage of aqueous humor. Subconjunctival scar tissue over the flap was dissected with the needle. In the case of a good filtration through the scleral flap, the sunconjunctival space then filled with aqueous humor and the bleb re-established. In the case of additional scarring tissue on the edges of the scleral flap, the needle was further advanced towards and through its margins. Fibrotic tissue was dissected, the flap was reopened, and the needle was advanced under the flap through the trabeculectomy opening in the anterior chamber. In most of these cases, the anterior chamber flattens and the eye becomes hypotonic. When the surgeon observed such a development, one or more sutures (10–0 nylon with cutting needle, Type CU-8, Circle: Bi-Curve; Alcon, Freiburg, Switzerland) were placed transconjunctivally through the scleral flap, adjacent sclera und were tied over the conjunctiva in order to tighten its margins to the adjacent sclera again, restore IOP and deepen the anterior chamber.

One to three sutures, according to the estimation of the surgeon for the intraoperative IOP, were placed and knotted tightly over the conjunctiva. The sutures move slowly through the conjunctiva in the following days, penetrate it and reach the subconjunctival space. This causes a relative relaxation of the elastic sutures, a decrease in the tension applied to the scleral flap, and thus a smooth reduction of the IOP. In the case of a persistently high IOP, these sutures can be removed either manually, if the suture is reachable over the conjunctiva (in the first postoperative days), or by argon laser suturolysis, if the suture is already in the subconjunctival space (in the later postoperative period).

All bleb surgeries were performed at the Department of Ophthalmology, University Medical Center in Mainz, by the same surgeon (NP). After surgery, all patients remained hospitalized for about one week and received daily slit-lamp and fundus examinations. All patients received the same postoperative management according to the standards of our hospital, taking into consideration the IOP and the morphology of the bleb: antibiotic and steroid eye drops plus subconjunctival injections of 5 Fluorouracil (5-FU), bulbus massage, etc.

The needling group (group 1) included 12 male and 11 female Caucasians, whose mean age was 72.78 ± 7.38 years. 15 patients had primary open-angle glaucoma, four pseudoexfoliative glaucoma, one patient pigment dispersion glaucoma, one patient juvenile glaucoma, one patient normal-tension glaucoma, and one patient secondary glaucoma.

The needling plus transconjunctival scleral flap sutures group (group 2) included 11 male and 12 female Caucasians with an average age of 72.52 ± 6.96 years. 13 patients suffered from open-angle glaucoma, seven from pseudoexfoliative glaucoma, two from normal-tension glaucoma, and one from pigment dispersion glaucoma.

After discharge from the clinic, patients were routinely examined on an outpatient basis for two years. Data collected on visual acuity, visual field examinations using white on white perimetry (Octopus^®^, Haag-Streit^®^, Wedel, Germany) slit-lamp biomicroscopy, Goldmann applanation tonometry, and confocal -Scanning-Laser Ophthalmoscopy by Heidelberg Retina Tomography (HRT^®^, Heidelberg Engineering, Heidelberg, Germany) were used for analysis. For the estimation of the course of glaucoma we used the Rim Area Change of the Moorfields Regression Analysis tool of HRT^®^.

Our major interest in this study was the long-term results of our modification of the standard needling procedure by adding transconjunctival scleral flap sutures. Since we already found a lower incidence of hypotony at the early postoperative period in a preceeding study, we now wanted to investigate the influence of our combined procedure on bleb survival, IOP development and progression of glaucoma in the long-term.

Descriptive statistics were performed by means and standard deviation. P values lower than 0.05 were considered statistically significant. Groups were compared for different variables in single time points using Mann-Whitney U-test because of the lack of a normal disturbution of the results.

Microsoft Excel 8.6 (Microsoft, Redmond, Washington, USA) and SPSS for Windows (version 16.1; SPSS Inc, Chicago, Illinois, USA) were used for the analysis.

## Results

### Intraocular pressure

**Fig 1:**
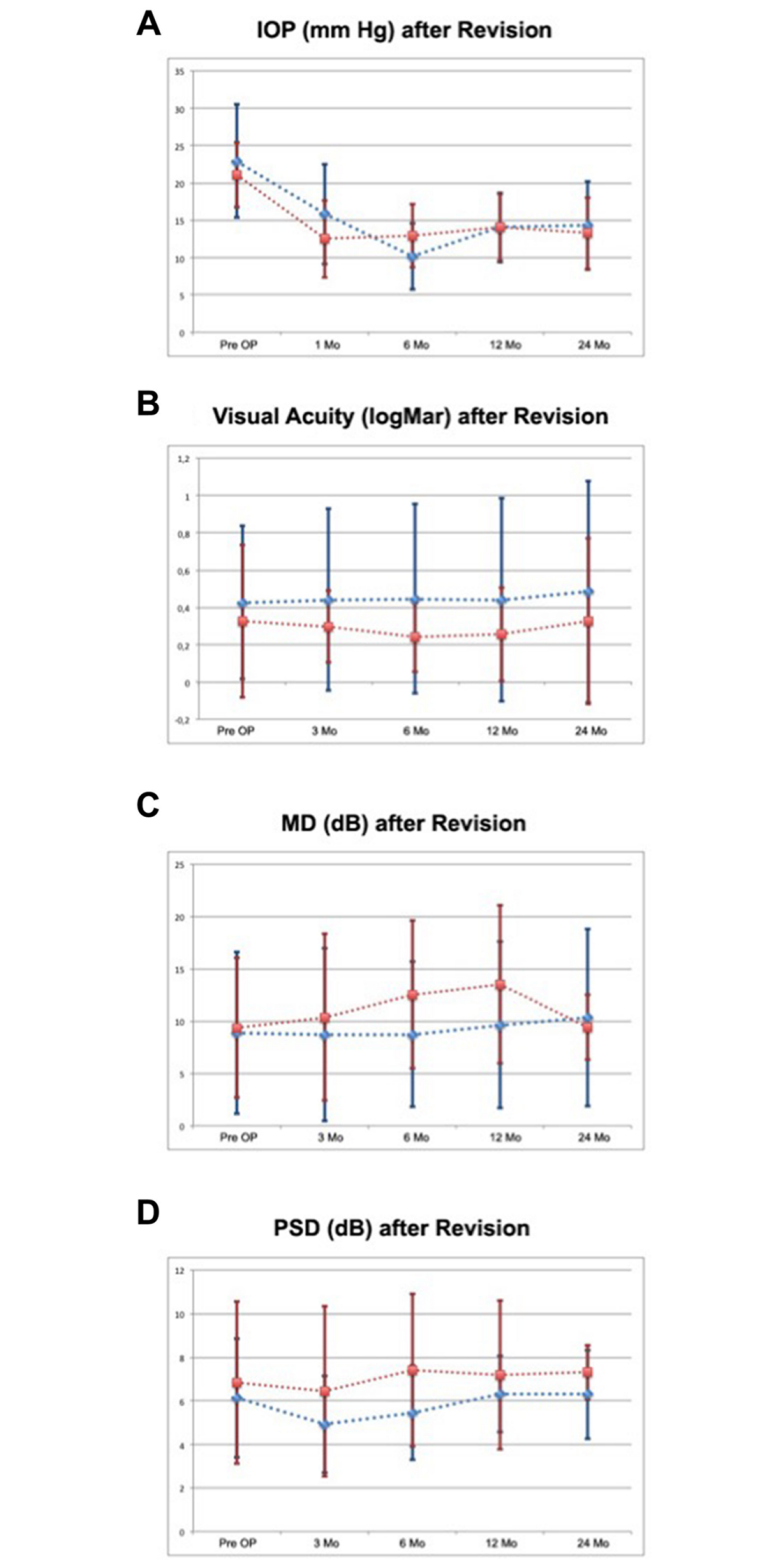
Courses of the intraocular pressure (A), visual acuity (B), mean deviation (C) and pattern standard deviation (D) for two years after the bleb revision in both study groups. The group, which received single needle procedure, is shown in blue, while the group, which received a combined procedure of needling with transconjunctival scleral flap sutures, is shown in red.

After the initial trabeculectomy and prior to the revision operation the mean intraocular pressure (IOP) statistically did not differ significantly between the two groups of the study (P = 0.59). IOP was 22.96 ± 7.57 mmHg in group 1 (needling) and 21.09 ± 4.37 mmHg in group 2 (needling plus transconjunctival flap sutures). The long-term follow up showed a similar IOP behaviour between the two groups (Table 1A and Fig 1A). After two years, the IOP was 14.33 ± 5.91 mm Hg in group 1 and 13.27 ± 4.78 mm Hg in group 2 (P = 0.57).

**Table 1:** Intraocular pressure (A), visual acuity (B), mean deviation (C) and pattern standard deviation (D) in the visual fields examination before bleb revision and at specific time points within the following two years in both study groups. Results are presented as means with standard deviation. P values for the comparison between the study groups are also shown.

**A. IOP (mmHg)**	Needling Group	Needling + Sutures	P Value
Preoperative 3 Months post OP 6 Months post OP 1 Year post OP 2 Years post OP	22.96 ± 7,57 15.86 ± 6.69 10.20 ± 4.47 14.08 ± 4,66 14.33 ± 5.91	21.09 ±4.37 12.53 ± 5.18 12.94 ± 4.25 14,10 ± 4,48 13.27 ± 4.78	0.59 0.13 0.12 0.92 0.57
**B. Visual Acuity (LogMar)**	Needling	Needling + Sutures	P Value
Preoperative 3 Months post OP 6 Months post OP 1 Year post OP 2 Years post OP	0.43 ± 0,41 0.44 ± 0.49 0.45 ± 0.51 0.44 ± 0.55 0.49 ± 0.60	0.33 ± 0.41 0.3 ± 0.19 0.24 ± 0.18 0.26 ± 0.25 0.33 ± 0.45	0.11 0.57 0.29 0.46 0.65
**C. Visual fields, MD (dB)**	Needling	Needling + Sutures	P Value
Preoperative 3 Months post OP 6 Months post OP 1 Year post OP 2 Years post OP	8.87 ± 7.73 8.72 ± 8.25 8.75 ± 6.93 9.65 ± 7.96 10.32 ± 8.49	9.37 ± 6.69 10.36 ±37.96 12.57 ± 7.05 13.55 ± 7.55 9.43 ± 3.09	0.71 0.60 0.25 0.19 0.93
**D. Visual fields, PSD, (dB)**	Needling	Needling + Sutures	P Value
Preoperative 3 Months post OP 6 Months post OP 1 Year post OP 2 Years post OP	6.14 ±2.73 4.93 ±2.22 5.47 ± 2.18 6.3 ± 1.75 6.3 ±2.05	6.83 ±2.22 6.43 ±3.89 7.40 ± 3.50 7.17 ± 3.41 7.32 ± 1.25	0.74 0.44 0.25 0.87 0.25

### Bleb survival/Need of revision or medication

The success of both approaches in terms of the bleb survival was as follows: in five patients in the needling group (21.7%) and in three patients in the needling plus scleral flap sutures group (13%), the revised bleb needed further surgical intervention (P = 0.27). In some other cases, medication alone was enough to maintain the target IOP: Four patients in the needling group (17.4%) and three patients in the needling plus scleral flap sutures group (13%) were treated with IOP lowering drops (mean 0.1 in both groups, P = 0.94). No adverse effects such as leakage, oozing, blebitis or endophthalmitis were observed in both groups.

### Visual acuity

Before revision, visual acuity was 0.43 ± 0.41 logMAR in group 1 and 0.33 ± 0.41 logMAR in group 2 (P = 0.11). In the long term, the development of visual acuity was similar between the two study groups (Table 1B and Fig 1B). Two years postoperatively, it was 0.49 ± 0.60 logMAR in group 1 and 0.33 ± 0.45 logMAR in group 2 (P = 0.65).

### Visual fields

Mean deviation (MD) and pattern standard deviation (PSD) before and after revision surgery in four consequent examinations at several time points are presented in Table 1C and 1D and Fig 1C and 1D. No statistically significant difference could be observed between the two groups in any time point of the study.

### HRT

Optic nerve head imaging with the Heidelberg Retina Tomograph (HRT) system before and two years after bleb revision showed no significant changes in either group of the study. The change in the rim area of the optic nerve was 0.01 ± 0.26 mm^2^ in group 1 and 0.05 ± 0.18 mm^2^ in group 2 (P = 0.289)

## Discussion

In this study we present and compare the long-term survival rates of the bleb after the standard needling procedure and the modified procedure with additional transconjunctival scleral flap sutures. Our study offers several interesting new findings:First of all, the method of needling is a reasonably successful procedure for the restoration of function in encapsulated blebs. Second, we showed that adding transconjunctival scleral flap sutures to the standard needling procedure, in order to avoid early postoperative hypotony, had no negative effect in terms of the long term regulation of IOP. Third, the lower intraocular pressure achieved through the revision surgery (either needling alone, or combined with transconjunctival scleral flap sutures) stabilized glaucoma within the follow-up period.

Our aim for the modification of the standard needling procedure by adding transconjunctival scleral flap sutures was to find a way to prevent early postoperative intraocular hypotony, which is reported to occur in 15–30% of cases after needling procedure.[8]. Postoperative hypotony is a well-known risk factor for developing suprachoroidal hemorrhage, a threatening complication, which goes along with poor visual outcomes [16].

In a previous study from our group, we were able to show that the addition of transconjunctival scleral flap sutures to the standard needling procedures lowers the incidence of postoperative hypotony and may protect patients from complications, including choroidal haemorrhage [13].

Although the results of this first study were very convincing regarding the immediate postoperative period showing a statistically significant higher IOP one week after surgery in the needling plus scleral flap sutures group, the long-term effects of additional sutures on the success of the bleb revision remained in question: Higher IOPs directly after surgery might have resulted into IOPs over our therapeutic goal range later in the follow-up. It was highly plausible that additional sutures might behave as a foreign body, thereby possibly activating and attracting fibroblasts and stimulating a cicatrization process. This would then easily lead to a secondary failure of the bleb and a rise in IOP. Even after the standard procedure such a development is not uncommon.

Over a period of two years after a single needling revision for encapsulated blebs, complete success of the procedure, defined as no necessity for additional measures for maintaining the target IOP, was achieved in 61% of the treated patients. If we also consider patients who maintained an adaequate IOP with IOP lowering medication, this percentage increased to 78% (qualified success). The results of the single needling procedure as performed in our department are in overall agreement with those of other retrospective studies of bleb revisions (50% to 80%) [7–9, 17].

Over the period of two years the percentage of patients with complete success after receiving our modified procedure with needling and transconjunctival sclerap flap sutures was 73%. Furthermore, the qualified success of our modified procedure increased to 87% of patients. Thus, the success rates of the bleb revision were similar between the two groups of our study.

Considering these results, it appears to us that the stimulation effect of additional scleral flap sutures on the scarring procedure of the bleb is overall not significant. In particular, after passing through the conjunctiva into the subconjunctival space of the bleb, we believe that the sutures do not show such an effect at all. Such a view is also supported by other studies in which different suturing models for the scleral flap in the standard trabeculectomy procedure were compared to each other: The number of the sutures was irrelevant to the success of the surgery [18], thus the scarring effect of a subconjunctival suture may be small or even absent. Regarding the very early postoperative time, during which the scleral flap sutures are still on or within the conjunctiva, a relative hyperemia of the bleb is obvious, as a sign of incipient scarring. As was shown in our previous publication, this can be managed through a more aggressive postoperative management of these patients with animetabolites. Moreover, this did not cause any adverse effects such as leakage, oozing, blebitis or endophthalmitis.

We therefore claim that the addition of transconjunctival scleral flap sutures to the needling procedure in order to prevent ocular hypotony on the short-term does not negatively impact the effectiveness of the revision on IOP and bleb survival in the long-term.

One limitation of our study is the fact that is was not randomized, as transconjunctival scleral flaps were placed only in intraoperatively hypotonic eyes. On the other hand, we regard this limitation as a positive indicator for the stronger efficacy of our approach: the modified procedure was only applied in cases where patients were susceptible to hypotony and even then, the course of intraocular pressure was similar to that after a standard procedure. A randomized study of cases with hypotony during the operation could reveal even greater advantage of the application of transconjunctival scleral flap sutures.

In light of the results of this study, we argue that the modification of the standard needling procedure with the addition of transconjunctival scleral flap sutures is effective: first, to prevent early ocular hypotony and second, to maintain the target IOP in the long-term. Our current approach towards an encapsulated bleb after trabeculectomy with high IOP is the following: after the standard needling procedure, the intraoperative aquous outflow and IOP are evaluated. In case of overfiltration with low IOP and flat anterior chamber, one to three transconjunctival scleral flap sutures are applied until the eye retains its pressure. The modified procedure is also favoured in the presence of risk factors for hypotony and subchoroidal hemorrhage, such as high myopia, hyperopic short eyes, and previous vitreoretinal surgery.

## Supporting information

S1 Excel Intraocular pressure. (XLSX)

S2 Excel Visual acuity. (XLSX)

S3 Excel Mean deviation. (XLSX)

S4 Excel Pattern standard deviation. (XLSX)

S5 Excel Rim area change. (XLSX)
